# Smear-Positive Tuberculosis Prevalence and Associated Factors among Pregnant Women Attending Antinatal Care in North Gondar Zone Hospitals, Ethiopia

**DOI:** 10.1155/2019/9432469

**Published:** 2019-03-03

**Authors:** Adugna Berju, Belete Haile, Seleshe Nigatu, Araya Mengistu, Girma Birhan

**Affiliations:** ^1^Department of Veterinary Epidemiology and Public Health, College of Veterinary Medicine and Animal Sciences, University of Gondar, P.O. Box 196, Gondar, Ethiopia; ^2^Department of Para Clinical Studies, College of Veterinary Medicine and Animal Sciences, University of Gondar, P.O. Box 196, Gondar, Ethiopia

## Abstract

Tuberculosis is an ancient infectious disease that remains a threat to public health around the world. It is a contagious airborne disease caused by *Mycobacterium tuberculosis* complex. In high tuberculosis burden countries, the prevalence of tuberculosis was 10-fold higher in the HIV-infected mothers than that in those not infected with HIV. However, little is known about the burden of tuberculosis (TB) and associated factors in women of reproductive age in most resource poor countries. Therefore, this study aims to investigate prevalence of smear-positive TB and factors associated in pregnant women attending antenatal care in North West, Ethiopia. An institution-based cross-sectional study was conducted in three governmental hospitals of the North Gondar Zone, and a total of 1272 pregnant women attending antenatal care were included. Data were collected by trained personnel's using a pretested and structured symptom screening questionnaire; then, spot-morning-spot sputum samples were collected from those pregnant women who had two or more weeks of cough, and sputum smear was done by using a light-emitted diode fluorescent microscope. 99% of the pregnant women visited the hospitals for antenatal care. The prevalence of smear-positive tuberculosis was 864/100,000 population, and HIV positivity (AOR = 7.24; 95% CI: (2.01–26.03)), urban residence (AOR = 2.28; 95% CI: (1.419–3.158)), and family history of TB (AOR = 2.12; 95% CI: (1.371–3.451)) were significantly associated with smear-positive tuberculosis. In this study, the prevalence of smear-positive tuberculosis was found to be higher than that in other community-based studies in the country. Therefore, health education, targeted screening of pregnant women for TB, and collaboration of TB-HIV clinic with antenatal care clinic should be implemented in the area. Further research should also be conducted for better understanding of the magnitude of tuberculosis in females of reproductive age.

## 1. Introduction

Tuberculosis is an ancient infectious disease that remains a threat to public health around the world [1]. Tuberculosis (TB) is a contagious airborne disease caused by *Mycobacterium tuberculosis* complex [2]. According to the recent WHO report, TB is the greatest cause of death of people living with HIV and ranks alongside HIV as a top infectious disease killer. Even at the moment after the accessibility of successful drugs for extra half a century, TB is a major cause of global morbidity and mortality. The WHO [2] report indicated that there were an estimated 10.4 million new TB cases worldwide, of which 6.2 million were men, 3.2 million were women, and 1 million were children.

Tuberculosis is most common during the woman's reproductive years and is a major cause of maternal child mortality [3]. In pregnant women living with HIV, increase in the risk of maternal and infant mortality by tuberculosis is almost 300% [2]. In high burden countries, the rate of active tuberculosis ranges from 0.7% to 7.9% among HIV-positive women and is as high as 11% if they are positive for tuberculin skin test [3, 4]. In developing countries, the TB prevalence was 10-fold higher in the HIV-infected mothers than that in those not infected with HIV [5].

Tuberculosis is a disease of poverty affecting vulnerable groups and mainly affects women when they are economically and reproductively active [2]. Once infected, women of reproductive age are more susceptible to developing TB than men of the same age as stated by the World Health Organization [6]. The disease is a significant contributor to maternal mortality and is among the three leading causes of death among women aged 15–45 years in high burden areas [7].

The burden of tuberculosis in pregnant women is substantially high [8], and data from sub-Saharan African countries show the consequence of *Mycobacterium tuberculosis* infection as a main cause of maternal mortality, especially in the context of HIV coinfection [9, 10].

Ethiopia is categorized among the 30 high burden countries (HBC) in the world with an estimated incidence rate of 192 new TB cases/100,000 populations [2]. In the country, smear-positive tuberculosis is found to be more prevalent in females than males [11–13].

Maternity services provide a unique opportunity for tuberculosis screening and subsequent follow-up [8]. Similarly, antenatal care in Ethiopia is a vital spot contact of pregnant women to assess and access regular screening for TB in pregnant women, and it is not ordinary practice in many settings which leads hindrance in diagnosis, maternal mortality, and child mortality. However, there is limited information on the prevalence of smear-positive tuberculosis and associated risk factors which contribute to the burden of tuberculosis in pregnant women of Ethiopia. Therefore, the aim of this study was to determine the prevalence of smear-positive tuberculosis and associated risk factors among pregnant women attending the antenatal care clinics of North Gondar Zone Hospitals in Ethiopia.

## 2. Materials and Methods

### 2.1. Study Design and Setting

Institution-based cross-sectional study was employed among pregnant women attending antenatal health care clinics of North Gondar Zone government hospitals, North West Ethiopia. The zone has an estimated total population of 3,285,351, and the zone is divided into 24 woredas, four city administrations, and 576 kebeles. According to the North Gondar Zone and Economic Development Department (2012), the zone has a total of 3 hospitals, 132 health centres, and 566 health posts with an overall 76% health coverage. In addition to the three governmental hospitals, there were private health centres providing antenatal health care service for pregnant women, and the service coverage was 71%.

### 2.2. Study Population

The study population was pregnant women who attended antenatal care from December 2015 to April 2016 in the selected governmental hospitals of North Gondar Zone.

#### 2.2.1. Inclusion and Exclusion Criteria

All pregnant women of 18 years of age or older presenting to the hospitals for ANC service were eligible to participate. Women who were unable to provide verbal consent and critically ill with other obstetrics case were excluded.

### 2.3. Sample Size Determination and Sampling Procedure

**Figure 1:**
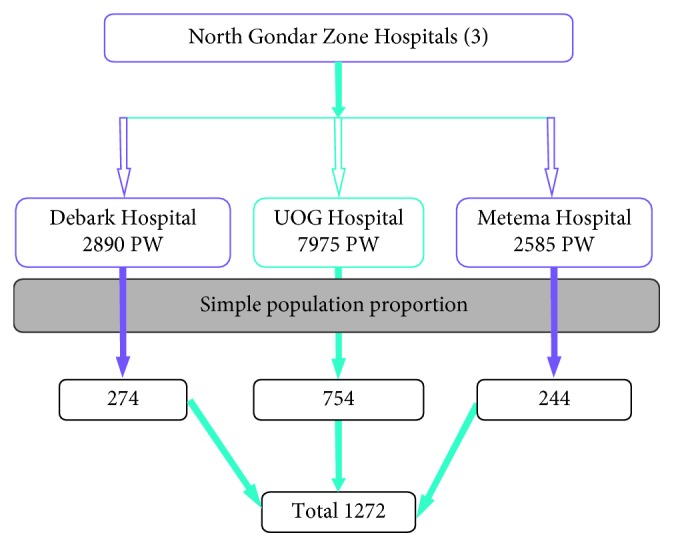
Schematic representation of sampling procedures on prevalence of smear-positive TB and associated factors among pregnant women in Northwest Ethiopia.

Three governmental hospitals in the study area were taken purposively, and the distribution of estimated sample size to each hospital was determined based on simple population proportion formula. Accordingly, from the three hospitals, namely, University of Gondar (UOG) Referral Hospital, Debark Hospital, and Metema Hospital, a total 1272 study subjects were sampled as described in Figure 1. The systematic random sampling technique was used to select every other woman who was available at the time of data collection and express their willingness to be included in the study.

### 2.4. Data and Specimen Collection

Data were collected by four trained nurses and three laboratory technicians using a pretested and structured symptom screening questionnaire among women coming to attend their antenatal care in the given hospitals. After obtaining written informed consent, each eligible pregnant woman was interviewed and maternal data including age, marital status, occupation, residence, family history of TB, other illnesses like HIV-AIDS and diabetics, and educational levels (no formal education, 1–9 grade: primary education, 10–12 grade: secondary education and diploma and above tertiary education) were obtained. Two spot-morning-spot-sputum samples for each TB-suspected pregnant woman having cough of at least 2 weeks' duration were collected using a labelled sputum container. Efforts were made to ensure a high quality of sputum by appropriate orientation of participants on how to produce sputum from lungs and to produce at least 3 ml of sputum, and supervision was made by laboratory workers. Then, the sputum sample was prepared on slide immediately as soon as possible for each participant at the day of collection. Sputum-smear microscopy using a light-emitting diode (LED) fluorescence microscopy was done in all TB laboratories of hospitals following the Partec GmbH manufacturer's procedure, and the results were interpreted accordingly. Two slides were stained for every sample, and the slides were read by two experienced laboratory technologists separately. For discordant results, a third expert microbiologist read the slides and the reports of the third reader were taken as final. Smear positivity was defined as the presence of at least one positive smear result using the LED microscopy.

### 2.5. Statistical Analysis

Data entry and cleaning was carried out using the Epi Info version 6 statistical software and analyzed by SPSS software package version 20.0. Descriptive statistics, such as frequency distribution, mean, and percentage, were employed for most variables. Backward stepwise binary and multiple logistic regression analysis was done to assess the relative importance of the explanatory variables to the independent variable. *P* value less than 0.05 was considered significant and the estimation of odds ratio (OR) with 95% confidence interval (CI) was used to test the statistical significance of variables.

### 2.6. Ethical Consideration

The study protocol was reviewed and approved by the Institutional Review Board of the University Gondar, Institute of Public Health, College of Medicine and Health Sciences. The hospital managers and administrators of the hospitals in the study areas were consulted, and permission was obtained prior to data collection. Written informed consent was obtained from each study subjects, and the purpose and benefits of the study were explained to the respondents. Confidentiality of the information was maintained throughout by excluding names as identification in the questionnaire and keeping their privacy during the interview by interviewing them alone.

## 3. Results

### 3.1. Sociodemographic Profile

A total of 1272 pregnant women aged ≥18 years were screened for tuberculosis through a symptom interview questionnaire. The mean age of respondents was 27.5 (±SD 5.2) years ranging from 18 to 43 years. In the present study, 37.4% of respondents had no formal education, and as to residence, 930 (73.1%) and 342 (26.9%) lived in urban and rural areas, respectively (Table 1).

### 3.2. Prevalence of Smear-Positive Tuberculosis

**Figure 2:**
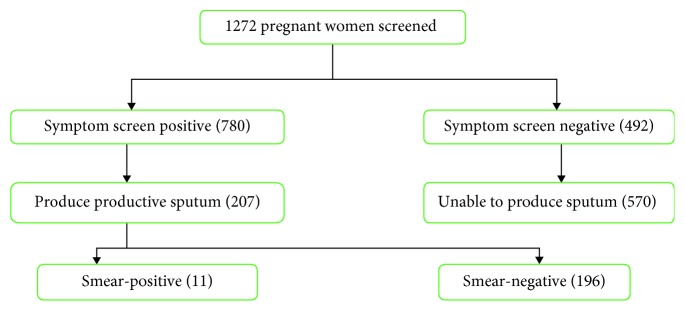
Flow chart for TB symptom screening in pregnant women.

As indicated in Figure 2, out of 1272 pregnant women who were screened for pulmonary tuberculosis through symptom interview, only 207 pregnant women produced productive sputum for microscopy examinations.

Of the total of 1272 TB symptom screen positive pregnant women, only 207 individuals produced productive sputum for microscopy examination. Of which, 11 (5.3%) patients had smear-positive results. Thus, the prevalence of new smear-positive TB in this study was 864 per 100,000 in people ≥18 years (95% CI: 0.004–1.3). As indicated in Table 2, higher rates of smear positivity were observed among HIV-positive individuals (AOR: 7.24, 95% CI (2.01–26.03)), followed by urban residence (AOR: 2.28, 95% CI (1.419–3.158)) and previous exposure of family history of tuberculosis (AOR: 2.12, 95% CI (1.371–3.451)).

The bivariate analysis revealed that family size, diabetics, residence, HIV infection, previous exposure, and family contact of tuberculosis were associated with smear-positive tuberculosis in pregnant women. However, only residence, HIV infection, and previous exposure of family contact were significantly associated with smear-positive tuberculosis and multivariate logistic regression analysis has been employed for controlling the effect of confounding factors (Table 2). Moreover, the respondents from the urban area were 2.28 times more likely to have smear-positive TB than women from rural residence (AOR: 2.28, 95% CI (1.419–3.158)).

The present study also showed statistically significant association between smear-positive tuberculosis and HIV-infected pregnant women. Women who are HIV positive were 7.24 times more likely to have smear-positive tuberculosis compared to those of HIV-negative patients (AOR: 7.24, 95% CI (2.01–26.03)).

## 4. Discussion

In this institutional-based cross-sectional study design, an overall 864/100,000 population smear-positive tuberculosis prevalence was found among the pregnant women. Almost an equivalent active tuberculosis case was reported in pregnant women presented to ANC in Soweto, South Africa, i.e., 889 per 100,000 populations [14]. Our finding was higher compared to the other studies where the prevalence of tuberculosis in Malawi pregnant women was 384 per 100,000 populations [15] while it was smaller in Tanzania (3.8%) [16, 17].

To our best knowledge, this is the first study showing the prevalence of smear positivity of tuberculosis in pregnant women of Ethiopia attending antenatal clinic. Many study findings reported that the burden of TB in pregnant women is high and has adverse impacts on perinatal and infant outcomes [4]. Surprisingly, our study also showed relatively higher rate of smear-positive tuberculosis in pregnant women. This high prevalence could pose problems to TB control in the children, and their family as tuberculosis can transmit vertically and horizontally to their babies.

This study also showed that previous exposure or family contact has a significant association with smear tuberculosis positivity during pregnancy. This finding is in agreement with the Ethiopian Ministry of Health TB/HIV and Leprosy guideline [18], describing that individuals who have close contact with smear-positive pulmonary tuberculosis are more likely to develop tuberculosis.

A symptom-screening test conducted in Kenya [19] showed high association between pulmonary TB with previous history, family history, and contact with other tuberculosis-infected persons. This may be because women who were exposed before or had contact with their families or other infected persons may develop active tuberculosis while they become pregnant and get immune-compromised.

Being HIV positive increases susceptibility to infection with *M. tuberculosis*, the risk of progression to TB disease, and the incidence and prevalence of TB [20]. It also increases the likelihood of re-infections and relapses of TB [18]. The lifetime risk of HIV-positive individuals to develop TB is 20–37 times greater than that of HIV-negative individuals. In our cross-sectional study, women with HIV seropositive have 7.24 times higher probability of smear positivity than HIV-uninfected pregnant women (AOR = 7.24; 95% CI: (2.01–26.03)). This is consistent with the studies in Malawi by Chanyuka et al. [15], South Africa by Hoffmann et al. [16], and Tanzania by Sheriff et al. [21], which explained that HIV-seropositive pregnant women had higher prevalence of tuberculosis infection than HIV-negative pregnant women. The association might be due to possible reasons that HIV induced immune suppression and increase the risk of re-activation of latent tuberculosis.

In the present study, pregnant women from urban areas were two times and more likely to be infected with tuberculosis than women from rural areas (AOR = 2.12, 95% CI (1.371–3.451)). This is in line with the study conducted in North West Ethiopia and a study in Rajshahi City of Bangladesh [22, 23]. However, higher prevalence of smear-positive TB was reported in rural areas of Northwest Ethiopia and Zambia compared with that in urban areas [12, 24].

In many studies, increased age has been mentioned as an associated risk factor for TB progression and reactivation [12, 25]. However, in our study, age was not found to be associated with smear-positive tuberculosis in pregnant women. This may be because study participate in our study was with the mean age of 27.5 (±SD 5.2) younger than the mean age of the above community-based studies.

In our current study, diabetics has a strong association with bivariate analysis but not with multivariate. Similarly, a study conducted in Tanzania [26] showed diabetics as a strong risk factor for pulmonary TB. Although diabetics revealed strong association in the bivariate, it remains insignificant. As our current study, Aliyu et al. [26] reported the association of diabetes and pulmonary tuberculosis depend on the HIV status of patients.

## 5. Conclusion

The prevalence of smear-positive tuberculosis in pregnant women in the North Gondar Zone Hospitals is relatively high. Risk factors like urban residence, HIV infection, and previous exposure/family history of tuberculosis had strong association with smear-positive tuberculosis in pregnant women. Thus, sufficient funding and proper implementation of screening program in pregnant women, especially HIV-positive pregnant women, have the potential to significantly enhance the ending of TB in Ethiopia. Furthermore, research targeting pregnant women and developing locally appropriate screening program in every ANC service provider health institution and TB/HIV clinic and collaboration of the referral hospitals would enhance the countries TB control strategies.

## Figures and Tables

**Table 1:** Sociodemographic characteristics of pregnant women in Northwest Ethiopia (*n*=1272).

Variables	Frequency	Percentage
Age		
18–25	478	37.6
26–35	682	53.6
>36	112	8.8

Educational level		
No formal	476	37.4
Primary	216	17
Secondary	303	23.8
Tertiary	277	21.8

Marital status		
Single	52	4.1
Married	1184	93.1
Divorced	31	2.4
Widowed	5	0.4

Occupation		
House wife	705	55.4
Private work	326	25.7
Government employee	241	18.9

Residence		
Urban	930	73.1
Rural	342	26.9

**Table 2:** Prevalence of smear-positive TB detected by selected sociodemographic and clinical factors of pregnant women in North Gondar Zone (*n*=1272).

Variables	Positive for FM		Crude OR (95%)	Adjusted OR (95%)	*P* value
	Yes	No			
Family size					
≥5	8	325	7.68 (2.025–29.122)		
1–4	3	936	1		

Diabetics					
Positive	1	11	11.364 (1.338–96.546)		
Negative	10	1250	1		

Residence					
Urban	6	336	3.304 (1.002–10.89)	2.28 (1.419–3.158)^*∗*^	0.015
Rural	5	925	1		

Family TB history					
Yes	5	19	2.62 (1.70–4.06)	2.12 (1.371–3.451)^*∗*^	0.001
No	6	1242	1		

HIV status					
Positive	4	108	6.101 (1.758–21.170)	7.24 (2.01–26.03)^*∗*^	0.002
Negative	7	1153	1		

^*∗*^Statistically significant for tuberculosis at *P* value <0.05; FM: fluorescence microscopy; OR: odds ratio.

## Data Availability

The data used to support the findings of this study are available from the corresponding author upon request.
